# Control of Hepatitis B Virus by Cytokines

**DOI:** 10.3390/v9010018

**Published:** 2017-01-20

**Authors:** Yuchen Xia, Ulrike Protzer

**Affiliations:** 1 Liver Diseases Branch, National Institute of Diabetes and Digestive and Kidney Diseases, National Institutes of Health, Bethesda, MD 20892, USA; yuchen.xia@nih.gov; 2 Institute of Virology, Technische Universität München/Helmholtz Zentrum München, Munich 81675, Germany; 3 German Center for Infection Research (DZIF), Munich Partner Site, Munich 81675, Germany

**Keywords:** hepatitis B virus (HBV), cytokine, interferon, interferon-induced gene (ISG), cccDNA, therapy

## Abstract

Hepatitis B virus (HBV) infection remains a major public health problem worldwide with more than 240 million individuals chronically infected. Current treatments can control HBV replication to a large extent, but cannot eliminate HBV infection. Cytokines have been shown to control HBV replication and contribute to HBV cure in different models. Cytokines play an important role in limiting acute HBV infection in patients and mediate a non-cytolytic clearance of the virus. In this review, we summarize the effects of cytokines and cytokine-induced cellular signaling pathways on different steps of the HBV life cycle, and discuss possible strategies that may contribute to the eradication of HBV through innate immune activation.

## 1. Introduction

Despite the availability of effective vaccine, hepatitis B virus (HBV) remains a major public health threat with more than 240 million individuals chronically infected worldwide who are at high risk of developing liver cirrhosis and hepatocellular carcinoma (HCC) [1,2]. While >90% of young children are unable to clear the virus when exposed and become chronically infected, most adults infected with the virus recover. As a hallmark of self-limited HBV infection, individuals who resolved the infection during the acute phase have shown a strong and polyclonal T-cell response [3,4]. In contrast, a weak and exhausted T-cell response failing to control the virus has been observed in chronic infection [5]. 

Viral clearance during acute HBV infection is thought to be mediated by cluster of differentiation (CD)4+ and CD8+ cytotoxic T lymphocytes (CTLs) [6,7]. Cytotoxic T lymphocytes can directly recognize and kill infected hepatocytes. Pioneering work by Guidotti and Chisari, however, showed that immune cells also control the virus in a non-cytolytic fashion via the secretion of cytokines and other immune mediators [8]. 

Hepatitis B virus DNA was shown to largely disappear from the liver and the blood of acutely infected chimpanzees already before the peak of T-cell infiltration [8]. This demonstrated that non-cytolytic antiviral mechanisms not only contribute to viral clearance in acute viral hepatitis by purging HBV replicative intermediates from the cytoplasm, but also purge the HBV transcription template—covalently closed circular DNA (cccDNA)—from the nucleus of infected cells. For this non-cytolytic control of HBV, antiviral cytokines such as interferons (IFN) or tumor necrosis factor (TNF) secreted from T-cells and other sources play an essential role [9]). 

The antiviral activity of IFN-α is exploited for therapy. Currently, there are two classes of approved treatments for chronic hepatitis B: nucleos(t)ide analogs (NUCs) and IFN-α. Nucleos(t)ide analogs target the viral reverse transcriptase limiting virus replication, but they cannot clear HBV infection. Interferon-α is the only approved treatment that can cure HBV infection, although the response rate remains limited to 5%–20%, and side effects are often difficult to tolerate. As for other cytokines, the antiviral mechanisms of IFN-α are multiple and include immunomodulatory as well as direct antiviral effects.

In this review, we will focus on the antiviral effects of cytokines and summarize our current knowledge about the effect of different cytokines on the HBV life cycle. Finally, we will discuss potential therapeutic approaches to control HBV infection. 

## 2. Hepatitis B Virus Life Cycle

Hepatitis B virus is a small, enveloped DNA virus that specifically targets hepatocytes. Hepatitis B virus infection is initiated via interaction of the “a” determinant(s) with heparan sulfate proteoglycans (HSPG, low affinity), resulting in a large envelope protein being able to bind to sodium-taurocholate cotransporting polypeptide (NTCP, high affinity) facilitating viral entry [10,11]. After entry and uncoating of HBV, the viral capsid is transported to the nucleus, where the partially double-stranded relaxed circular DNA (rcDNA) genome is released and converted into cccDNA by host enzymes [12,13]. This cccDNA persists as a minichromosome and serves as a template for transcription of four viral RNAs through the cellular transcription machinery. A number of liver-enriched transcription factors and nuclear receptors have been shown to bind HBV promoter or enhancer elements and to be critical in activating and regulating HBV transcription [14]. The major open reading frames (ORFs) that are translated are as follows: the precore/core ORF, coding for the HBV core protein that form the capsid and the hepatitis B virus e antigen (HBeAg); the largest ORF encoding a multifunctional polymerase protein comprising reverse transcriptase, RNase H, and protein primer domains; three largely overlapping surface protein ORFs encoding the large (L), medium (M), and small (S) envelop proteins; and the smallest ORF encoding the regulatory X-protein. 

The longest, 3.5 kb pregenomic RNA (pgRNA) is encapsidated together with the viral polymerase. Inside the viral capsid, pgRNA is reverse transcribed into negative-strand DNA. The addition of a plus strand that remains incomplete results in the formation of the rcDNA viral genome. rcDNA-containing capsids are either transported back to the nucleus to establish a cccDNA pool or enveloped and released via multivesicular bodies as progeny virions [15]. 

## 3. Inhibition of Virus Entry by Cytokines

Virus entry into host cells is the starting point of a productive infection process. Inhibition of viral entry is thus a promising approach to control a virus. Targeting either of the two host factors HSPG or NTCP that are crucial for HBV entry is exploited by cytokines or cytokine-induced mediators.

By using HBV-infected HepaRG cells, we demonstrated that IFN-α-treated cells release factors restricting HBV entry [16]. These factors competed with the virus for binding to HSPG. This study revealed a novel antiviral mechanism of IFN-α: inhibiting HBV infection of neighboring cells by inducing soluble factors that bind to HSPG and block HBV attachment.

Recently, cholesterol-25-hydroxylase (CH25H) was identified as an interferon-induced gene (ISG) [17]. Cholesterol-25-hydroxylase converts cholesterol to 25-hydroxycholesterol (25HC), which inhibited entry of a number of enveloped viruses including HBV [18].

The cytokines interleukin (IL)-6 and IL-1β regulate NTCP expression and can thus inhibit HBV entry [19,20]. A recent study revealed that HBV entry was inhibited by up to 90% when cells were pretreated with IL-6, resulting in a strong reduction of cccDNA and HBsAg secretion [21]. In parallel, decreasing NTCP mRNA level led to a strong reduction in NTCP-mediated taurocholate uptake in a dose-dependent fashion. HBV entry and bile acid uptake were restored by lentiviral overexpression of NTCP. This indicated that IL-6 inhibits HBV entry through the downregulation of the viral entry receptor NTCP, most likely by inhibiting hepatocyte nuclear factor (HNF) 4α-mediated transcription [22].

## 4. Cytokine-Mediated Covalently Closed Circular DNA Degradation

Persistence of the viral transcription and replication template, the cccDNA minichromosome, is the major problem for antiviral therapy because the clearance of HBV requires elimination or long-term silencing of cccDNA to prevent reactivation of viral replication and relapse of infection. Recently, we showed that high doses of IFN-α are able to trigger non-cytolytic purging of cccDNA from infected primary human hepatocytes or HepaRG cells. Interferon-α activates the nuclear deaminase APOBEC3(A3)A that utilizes hepatitis B virus core protein to get access to cccDNA. This upregulation of A3A was confirmed in liver biopsies from IFN-α treated patients and chimpanzees. Deaminated cccDNA becomes prone to degradation by nucleases that are also IFN-regulated. Treatment of deaminated cccDNA with base excision repair (BER) enzymes uracil-DNA glycosylase (UNG) and apurinic/apyrimidinic endonuclease leads to cccDNA decay [23]. Interestingly, lymphotoxin-β receptor-agonists that induce the nuclear deaminase A3B in a nuclear factor κB (NFκB)-dependent fashion can activate the same pathway, resulting in cccDNA purging [23]. Furthermore, Bockmann et al. showed that type III IFN (IFN-λ) induce cccDNA degradation similar to type I IFN in HBV-infected HepaRG cells. Interferon-λ1 and IFN-λ2 efficiently suppress HBV replication and reduce intracellular cccDNA similar to IFN-α but with distinct kinetics [24].

The clinical relevance of these findings was validated by gene expression profiling of patient biopsies taken after a single dose of pegylated (PEG)-IFN-α [23] and more recently in patients with hepatitis B. The latter study compared BER gene expression and antiviral effects in patient blood and liver biopsy samples taken before and after PEG-IFN-α therapy. A3A and different BER genes were regulated by PEG-IFN-α treatment [25].

T-cell-derived cytokines IFN-γ and TNF-α also induce A3A and A3B in a synergistic fashion, an effect becoming obvious also during acute or fulminant hepatitis B [9]. Both IFN-γ and TNF-α interfered with cccDNA integrity and stability in HBV-infected primary human hepatocytes and HepaRG cells [9]. Rescue of cccDNA by A3A and A3B small interfering RNA proved IFN-γ and TNF-α induced cccDNA deamination and subsequent degradation [9]. Hepatitis B virus-specific T-cells were able to inhibit HBV infection and cause cccDNA loss without direct cell–cell contact, proving their non-cytolytic antiviral activity in HBV infection. Blockade of IFN-γ and TNF-α by neutralizing antibodies abrogated the non-cytolytic antiviral effect of T-cells and confirmed that these two cytokines are key to HBV inhibition [9]. Our results identified the molecular mechanism of how T-cells not only control HBV but even eliminate its persistence form in a non-cytolytic fashion. 

Qiao et al. showed that transforming growth factor (TGF)-β could induce cccDNA deamination and degradation in hepatocytes via activation-induced cytidine deaminase (AID) [26]. Accordingly, AID-mediated degradation of duck HBV (DHBV) cccDNA was described [27]. The effect elicited by TGF-β was abrogated when AID or the activity of UNG was blocked, indicating that AID-mediated deamination and the excision of uracil by UNG act in concert to degrade HBV cccDNA [26]. Similar to A3A, the interaction between AID and viral cccDNA was mediated by HBc [26]. 

## 5. Cytokine-Mediated Epigenetic Control of the Covalently Closed Circular DNA Minichromosome

Multiple evidence suggests that epigenetic modifications of HBV minichromosomes, such as histone modifications and DNA methylation, participate in regulating the transcriptional activity of HBV cccDNA. Histones and non-histone proteins either bind directly to the cccDNA or are indirectly recruited to viral minichromosomes through protein–protein interactions [28].

Belloni et al. showed that IFN-α inhibits HBV replication by decreasing transcription of viral RNA from the HBV cccDNA minichromosome in cell cultures and in humanized mice [29]. Interferon-α treatment reduced the binding of the transcription factors signal transducer and activator of transcription (STAT)1 and STAT2 to the IFN-sensitive response element on active cccDNA [29]. Interferon-α treatment also resulted in hypoacetylation of cccDNA-bound histones as well as active recruitment of transcriptional corepressors including histone deacetylases (HDAC) 1 to cccDNA [29]. 

Using a DHBV cell culture model, Liu et al. confirmed that IFN-α reduces the acetylation of H3K9 and H3K27 histone marks associated with cccDNA minichromosomes [30]. Moreover, DHBV cccDNA transcription was blocked by HDAC inhibitors, suggesting that DHBV cccDNA transcription may require HDAC activity [30]. 

A recent study conducted by Tropberger et al. demonstrated that IFN-α represses HBV by reducing active histone marks on the cccDNA minichromosome in HBV-infected primary human hepatocytes [31]. This effect can be recapitulated by treatment with a small epigenetic agent, C646, which specifically inhibits p300/CREB binding protein (CBP) histone acetyltransferases [31]. 

Similar observation was also reported for IL-6. Palumbo et al. treated HepG2 cells transfected with linear HBV monomers and HBV-infected NTCP-expressing HepG2 cells with IL-6 and found a reduction of cccDNA-bound histone acetylation paralleled by a rapid decrease in all HBV RNA transcripts without affecting cccDNA integrity [32]. Interleukin-1β, an inducer of IL-6, is able to silence cccDNA transcription via inhibitory NFκB binding to cccDNA [33,34]. The antiviral effect of IL-6 on HBV replication is additionally mediated by a reduction of transcription factor HNF4α [22] and by the redistribution of STAT3 binding from the cccDNA to IL-6 cellular target genes [32]. 

## 6. Transcriptional Control by Cytokines

Several cytokines have been shown to control HBV transcription through liver-enriched transcription factors. Using nuclear run-on assays, Uprichard et al. demonstrated a reduced transcriptional activity of the HBV genome in livers of HBV-transgenic mice that were either infected with a DNA virus or treated with polyinosinic/polycytidylic acid (poly I:C) inducing IFN-α and IFN-β [35]. Transcriptional repression of HBV by IFN-induced tripartite motif 22 (TRIM22) was reported by Gao et al.—TRIM22 inhibited HBV core promoter activity and thus HBV gene expression and replication in vitro and in vivo [36].

Interleukin-4 that is well known to induce differentiation of naive helper T-cells to T helper 2 cells, shows a direct antiviral effect on HBV [37]. Using luciferase reporter assays, Li et al. showed that IL-4 reduced activity of the HBV surface promoter II and core promoter by suppressing CAAT-enhancer-binding protein α (C/EBPα) [37]. 

Hosel et al. demonstrated that IL-6 suppresses expression of HNF1α and HNF4α, two major transcription factors determining HBV promoter activity, by activating mitogen-activated protein kinases c-Jun N-terminal kinase (JNK) and ERK and thus regulates HBV replication at transcriptional level in infected primary human hepatocytes [22]. Interleukin-1β was reported to suppress cccDNA transcription via hepatocyte dedifferentiation, which also resulted in the loss of HNF4α [33,34]. 

By using HBV plasmid-transfected hepatoma cells, Hong et al. reported that TGF-β specifically diminishes HBV core promoter activity, resulting in a reduction of pgRNA [38]. Similarly to IL-6 [22], TGF-β repressed HNF4α expression, and HBV replication could be rescued by ectopic expression of HNF4α [38]. Thus, IL-6, IL-1β, and TGF-β target HBV transcriptional activity and thus HBV replication through modulating the expression of the essential transcription factor HNF4α, while IL-4 seems to repress C/EBPα. 

## 7. Targeting Post-Transcriptional Steps

Several earlier studies revealed that cytokines mainly control HBV replication at a post-transcriptional level. Guidotti et al. used an HBV transgenic mouse model and demonstrated that adoptively transferred virus-specific T-cells can abolish HBV gene expression and replication in the liver without killing the hepatocytes [39]. This antiviral function was mediated by T-cells secreting IFN-γ and TNF-α. These two cytokines eliminate HBV nucleocapsid particles from the cytoplasm including their cargo of replicating viral genomes [39]. In addition, they destabilize HBV RNAs by releasing a cleavage-sensitive RNA motive [40].

The group of Chisari reported that IFN-β or IFN-γ treatment controls HBV replication in murine hepatocytes in cell culture and eliminate pgRNA-containing capsids from the cells [41]. Hepatitis B virus transgenic mice treated with poly I:C inducing an IFN-α/β response showed a 10-fold reduction of cytoplasmic pgRNA-containing capsids within 9 h [42]. Since there was no change in the abundance of HBV mRNA or the translational status of cytoplasmic HBV transcripts, they concluded that type I IFNs either inhibit the assembly of pgRNA-containing capsids or accelerate their degradation [42]. By using immortalized murine hepatocyte cell lines stably transfected with an inducible HBV replication system, they found that replication-competent pgRNA-containing capsids are not produced when the cells are pretreated with IFN-β before HBV expression is induced [43]. Furthermore, the turnover rate of HBV RNA-containing capsids was not changed after IFN-β or IFN-γ treatment under conditions in which pgRNA synthesis was turned off [43]. Similarly, IFN-α and IFN-γ reduced the amount of intracellular HBV nucleocapsids in murine hepatoma AML12 cells supporting inducible HBV replication [44]. An IFN-induced cellular antiviral response seems able to distinguish and selectively accelerate the decay of replication-competent HBV capsids but not empty capsids in a proteasome-dependent manner [44]. Interferon-λ is believed to inhibit HBV replication via the same pathway [45]. Taken together, these results suggest that IFNs prevent the formation of replication-competent HBV capsids. 

Similar findings were reported by Biermer et al. studying signaling pathways involved in the antiviral effect of TNF-α on HBV. Tumor necrosis factor-α inhibits HBV replication by disrupting capsid integrity via NFκB signaling [46]. 

A number of ISGs have been identified to be involved in post-transcriptional control of HBV replication. Interferon-α induced zinc finger proteins mediated viral RNA decay in vitro and in vivo [47,48]. Myeloid differentiation primary response protein 88 (MyD88), which can be induced by IFN-α, accelerated the decay of viral pgRNA [49]. Several IFN-induced cytosine deaminases including APOBEC3G (A3G) and AID-induced HBV genome hypermutation thus inhibit virus infection [50,51,52]. Furthermore, A3G is incorporated into the cytoplasmic HBV nucleocapsid to inhibit viral DNA replication in a deamination-independent fashion [53,54]. Myxoma resistance protein 1 (MxA) inhibited viral pgRNA encapsidation [55,56]. Thus, ISGs elicit a number of antiviral effects on HBV.

## 8. Prevention of Viral Protein Translation

IFN was described to inhibit protein translation of several viruses including human immunodeficiency virus, dengue virus, and hepatitis C virus (HCV) [57,58,59]. Park et al. reported that antiviral effect of IFN-α was impaired by knocking-down RNA-dependent protein kinase (PKR) through RNA-interference [60]. The intracellular level of viral capsids was reduced in a PKR-dependent manner, whereas HBV RNA per se was not affected. The authors concluded that PKR functions as a key mediator of IFN-α in opposing HBV replication, most likely through the inhibition of protein synthesis [60]. Mao et al. demonstrated that indoleamine 2,3-dioxygenase (IDO1), an IFN-γ-induced tryptophan degradation enzyme, efficiently reduced the level of intracellular HBV DNA without altering the steady-state level of viral RNA in transfected HepG2 cells [61]. This antiviral effect of IDO1 depended on the induction of tryptophan deprivation, preferentially inhibiting viral protein translation but not significantly altering global protein synthesis in the host cell [61]. Thus, ISGs are able to block protein synthesis of HBV.

## 9. Blockade of Viron Secretion

Tetherin is an interferon-induced host restriction factor that blocks the egress of a variety of enveloped viruses through tethering the budding virions on the cell surface via its membrane anchor domains. Yan et al. described that IFN-α treatment of HepAD38 cells, an inducible HBV replicating cell line, reduced HBV virion release via tetherin without altering intracellular viral replication or the secretion of HBV subviral particles and non-enveloped capsid [62]. Microscopy analyses demonstrated that tetherin colocalizes with HBV virions on the multivesicular body, which is the HBV virion budding site [62], indicating that IFN-induced tetherin may also act on HBV.

## 10. Summary and Prospective

A complete cure of hepatitis B can, in theory, be achieved by elimination of viral infection via inhibition of the viral replication intermediate, a complete decay of cccDNA, and a total blockade of reinfection. Current therapeutic goal of chronic hepatitis B is a functional cure of HBV infection comprising HBsAg loss, anti-HB seroconversion, and inactivation of cccDNA, but it is rarely achieved with treatments currently available [63]. Cytokines are believed to be involved in the non-cytolytic clearance of HBV during acute infection and during T-cell-mediated virus control, and their antiviral effects are approved in a variety of experimental models. 

As summarized in Table 1, different IFNs and proinflammatory cytokines show antiviral effects on HBV. Although some of the mechanisms have not been tested in primary human liver tissue or HBV-infected primary human hepatocytes, the cytokine-mediated antiviral effects are multifunctional and target several key steps of viral replication. In this respect, cytokine-based therapies could provide interesting approaches to achieve a functional cure or even the eradication of virus infection if the side effects can be managed. 

One option is to improve IFN-α treatment. It is well accepted that, although binding to the same receptors, different IFN-α subtypes mediate different biological functions and display distinct antiviral activities [64]. Interferon-α2b has been used to treat hepatitis B for more than 25 years [65]. However, it may not be the most potent IFN-α subtype [66]. Antiviral and side effects of other IFN-α subtypes on HBV infection should be further studied. The low response rates and significant side effects of IFN-α treatment may be overcome by a higher local concentration in the liver. PEGylation has been developed to counter this problem via extending the half-life of IFN-α and has been widely used in several clinical applications [67]. However, the high cost of in vitro chemical coupling, accumulating evidence on the immunogenicity of PEG and lacking biodegradability of the unnatural PEG polymer, which can lead to renal tubular vacuolation hamper its application [68,69]. Recently, a novel, longer acting IFN-α was generated via PASylation technology [70]. PASylated IFN-α showed strong receptor binding affinity without inducing an observable immunogenic footprint [71]. More importantly, PASylated IFN-α demonstrated superior efficacy against HBV replication in HBV transgenetic mice than IFN-α [72]. 

Besides IFN-α, other IFNs may also consider as potential treatments for HBV infection. IFN-β, which is used to treat multiple sclerosis, has been considered as an alternative. The efficacy of IFN-β for HCV infection has been extensively investigated [73,74,75]. These studies not only proved the efficacy of IFN-β as an HCV therapy, but also showed promising results in patients who had poor responses to IFN-α2b/ribavirin treatment. The effect of IFN-β on chronic hepatitis B needs to be investigated. 

Wu et al. showed that HBsAg-positive patients with stage 2–4 hepatic fibrosis achieved a significantly improved fibrotic score after nine months of IFN-γ treatment [76]. However, the majority of patients did not show a long-term benefit. Restricted expression of IFN-λ receptors is expected to positively impact on the toxicity profile of IFN-λ [77]. A Phase IIb clinical trial of PEG-IFN-λ on chronic hepatitis C patients showed good response rates and tolerability [78]. This result should encourage clinical trials in chronic hepatitis B patients. 

Activation of cytokine in the liver is another option for hepatitis B treatment. Small molecule toll-like receptor (TLR) agonists have been developed for this purpose. Promising results were obtained with TLR-7 agonists, GS-9620, that is able to induce IFNs and ISGs, in woodchucks and chimpanzees. In woodchucks, an oral application of GS-9620 leads to sustained viral load reduction, induced anti-HBs seroconversion, and a reduced incidence of hepatocellular carcinoma [79]. In chimpanzees, short-term oral administration of GS-9620 provided long-term suppression of serum and liver HBV DNA, and low doses were well-tolerated in chronic hepatitis B patients [80,81]. Its therapeutic effect, however, cannot be hepatocyte- or liver-specific due to TLR-7 expression profiles, and further clinical trials are needed to judge the suitability of TLR-7 agonists as hepatitis B therapeutics. 

The concept of immunomodulation through therapeutic innate immune activation has a substantial amount of experimental evidence. Translating the experimental results into effective novel therapies that minimize potential side effects could promote both the cytokine-mediated innate immune control of viral infection as well as restoration of adaptive immunity. In combination with other therapeutics, immune therapy may contribute to the eradication of HBV.

As elaborated above, T-cell-derived cytokines have distinct antiviral potential. They contribute to HBV control and elimination in a non-cytolytic fashion, which, in addition to the cytotoxic effect of T-cells, results in the deprivation of HBV infection [9]. Thus, the adoptive transfer of T-cells is a promising option for hepatitis B treatment [82,83,84]. Qasim et al. reported an adoptive transfer of engineered lymphocytes into a patient who had undergone liver transplantation for HBV-related HCC and carried tumor cells expressing HBsAg, which can be recognized by T-cells expressing an HBV-specific T-cell receptor [85]. They demonstrated that gene-modified T-cells survived in vivo, expanded, and mediated a reduction in HBsAg levels without exacerbation of liver inflammation or other toxicity [85]. This encourages the development of therapies restoring T-cell responses in chronic hepatitis B by therapeutic vaccination, adoptive T-cell transfer, redirection of T-cells, or the use of checkpoint inhibitors. 

## Figures and Tables

**Table 1:** Control of hepatitis B virus (HBV) by cytokines.

Targeted Step of Virus Life Cycle	Cytokines	Active Factors	References
HBV entry	IFN-α, IL-6 *	CH25H	[16,18,21]
Epigenetic control of cccDNA	IFN-α *, IL-6, IL-1β *	HDAC *, STAT1 *, STAT2 *, STAT3	[29,30,31,32,33,34]
cccDNA integrity	IFN-α *, IFN-γ *, IFN-λ, TNF-α *, TGF-β,	A3A *, A3B *, AID	[9,23,24,26,27]
Transcription of HBV RNAs	IL-4, IL-6 *, TGF-β, IFN-α *, IFN-β, IL-1β *	TRIM22	[22,33,34,36,38,49]
Post-transcriptional targeting of RNA and capsid stability	IFN-α, IFN-β, IFN-λ, IFN-γ, TNF-α, IL-1β	ZAP, MyD88, A3G, MxA, AID	[18,41,42,43,44,45,46,47,48,49,50,52,53,54,55,56]
Protein translation	IFN-α, IFN-γ	PKR, IDO1	[60,61]
Viron secretion	IFN-α	Tetherin	[62]

* Cytokine-mediated mechanisms that have been verified in primary human liver tissue or HBV-infected primary human hepatocytes. AID: Activation-induced cytidine deaminase; A3: APOBEC3; cccDNA: Covalently closed circular DNA; CH25H: Cholesterol-25-hydroxylase; HDAC: Histone deacetylases; IDO1: Indoleamine 2,3-dioxygenase; IFN: Interferon; IL: Interleukin; MxA: Myxoma resistance protein 1; MyD88: Myeloid differentiation primary response protein 88; PKR: RNA-dependent protein kinase; STAT: Signal transducer and activator of transcription; TNF: Tumor necrosis factor; TRIM22: Tripartite motif 22; ZAP: Zinc-finger antiviral protein.
